# Supplementary material for: Violent Content in Online Pornography Is Associated with Sexual Health of Women and Men
Source: Behav Sci (Basel). 2025 Nov 27;15(12):1634. doi: 10.3390/bs15121634 (PMC12729544; doi:10.3390/bs15121634)
Supplement: Supplementary file 1 [file behavsci-15-01634-s001.zip › behavsci-3935449-supplementary.pdf]

SUPLEMENTARY MATERIAL

Table S1

Pornographic practices consumed in the last 12 months by young adult women and men (18-35 years) living in Spain.

	**Viewed pornography in the last 12 months**
**Pornography without explicit violence**	
P1	Naked people without having sexual intercourse
P2	CONSENTED sexual intercourse WITHOUT THE USE OF FORCE
P3	Manga videos showing CONSENTED sexual intercourse AND WITHOUT THE USE OF FORCE
**Pornography with degrading/humiliating practices without physical violence**	
P4	Dominance and submission WITHOUT CAUSING PHYSICAL PAIN
P5	GAMES with semen and/or saliva and/or other fluids, on the body, face or mouth of one/many people
P6	Sexual intercourse where one person verbally insults or threatens another person
**Pornography with physical violence**	
P7	Sexual intercourse with a DRUNK, UNCONSCIOUS OR SLEEPING PERSON
P8	Manga videos showing sexual intercourse that is NOT CONSENTED, WITH USE OF FORCE, although in the end they both experience pleasure
P9	Sexual intercourse that is NOT CONSENTED, WITH USE OF FORCE, although in the end they both experience pleasure
P10	Sexual intercourse where one person PULLS THE OTHERS HAIR AND/OR SLAPS AND/OR HITS the other person
P11	Sexual intercourse where one person CHOKES the other person
P12	BDSM (bondage/dominance/submission/sadomasochism): BY spanking and/or whipping and/or wax, etc.

Table S2

Individuals diagnosed with a sexually transmitted infection in the last 12 months. by sex and the level of violence in the pornography practices viewed.

Sexually transmitted infections diagnosed in the last 12 months (na=21)	What type of pornography do you view?							
	Men (n=1249; n.n=50)				Women (n=736, na=35)			
	PNV (n=180)	PDH (n=231)	PPhV (n=788)	Total	PNV (n=154)	PDH (n=182)	PPhV (n=365)	Total
**HIV**	0	0	4	4	0	0	0	0
**Syphilis**	1	0	4	5	0	0	2	2
**Gonorrhea**	2	2	13	17	0	0	1	1
**Chlamydia**	1	1	7	9	0	2	7	9
**HPV**	0	1	11	12	7	4	13	24
**Genital or anal herpes**	0	0	6	6	0	2	4	6
**Hepatitis B or C virus**	0	0	1	1	0	0	1	1
**Other**	1	1	7	9	4	1	4	9
**Number of sexually transmitted infections diagnosed**								
0	178	227	745	1150	143	174	335	652
1	1	3	36	40	11	7	28	46
2	0	1	5	6	0	1	2	3
3	0	0	1	1	0	0	0	0
4	1	0	1	2	0	0	0	0
**Total**	**180**	**231**	**788**	**1199**	**154**	**182**	**365**	**701**

Note. n: number; n.a: no answer; HIV: Human Immunodeficiency Virus; HPV: Human Papillomavirus; PNV: Exclusively pornography without explicit violence; PDH: Pornography with degradation/humiliation practices, no physical violence; PPhV: Pornography with physical violence.
